# Chemical-enhanced thyroid cell detection using photonic crystal biosensors with phase-change materials

**DOI:** 10.1039/d5ra09237j

**Published:** 2026-02-19

**Authors:** Walaa M. Nouman, Fatma A. Sayed, Arafa H. Aly, S. E.-S. Abd El-Ghany, Samira M. Sallam

**Affiliations:** a Physics Department, Faculty of Science, Benha University Egypt; b TH-PPM Group, Physics Department, Faculty of Science, Beni-Suef University Egypt arafa.hussien@science.bsu.edu.eg arafaaly@auegypt.edu

## Abstract

This study proposes a one-dimensional defective photonic crystal (1D-DPC) biosensor based on the phase-change material Ge_2_Sb_2_Te_5_ (GST) for label-free detection of thyroid nodules in the visible range. The structure is designed as [(Diamond/Silica)^3^ GDG (Diamond/Silica)^3^], where G represents GST layers and D is an air-cavity defect. This design effectively distinguishes between benign and malignant thyroid nodules. The main advantage of GST is its reversible phase transition between amorphous and crystalline states, which changes the material's refractive index and absorption coefficient. By placing two GST buffer layers around the defect, the biosensor's resonance can be actively tuned, leading to higher sensitivity and better spectral selectivity. Transmission spectra were calculated using the transfer matrix method (TMM) in MATLAB for both normal and oblique TE-polarized incidence. Performance metrics—including sensitivity (*S*), quality factor (*Q*), and figure of merit (FOM)—were evaluated for both GST phases. At normal incidence and 220 nm defect thickness in the crystalline state, the biosensor achieved a sensitivity of 267 nm RIU^−1^ and a quality factor of 87.875, enabling clear differentiation between malignant and benign cases. The key novelty is comparing THz spectra of liquid and lyophilized plasma, where lyophilization enhances contrast and malignant-benign separation by reducing water masking effects. The results were compared with previous photonic crystal biosensors, showing that the GST-based design offers superior tunability and diagnostic accuracy due to its unique phase-change properties.

## Abstract 2

This study proposes a one-dimensional defective photonic crystal (1D-DPC) biosensor based on the phase-change material Ge_2_Sb_2_Te_5_ (GST) for label-free detection of thyroid nodules in the visible range.

## Graphical abstract

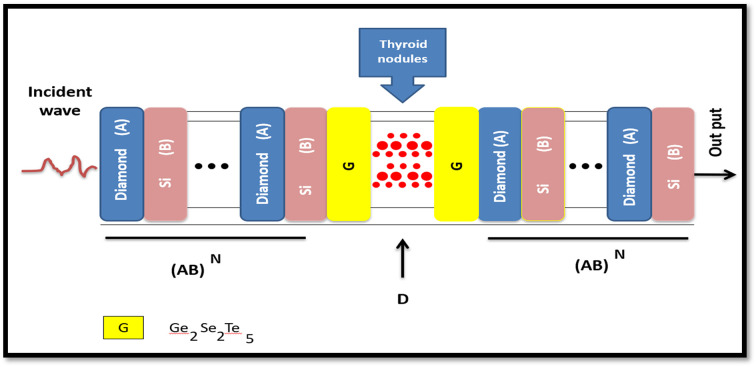

## 1 Introduction

Photonic crystals (PCs) are engineered materials composed of periodically arranged layers with contrasting dielectric properties, designed to regulate the propagation of electromagnetic waves within the structure. This periodic modulation of the refractive index leads to the formation of photonic band gaps (PBGs), which are specific frequency ranges where light propagation is prohibited due to Bragg scattering from the periodic interfaces. Owing to this unique ability to manipulate light, photonic crystals have found widespread applications in optical communication systems and advanced sensing technologies. By employing alternating high- and low-refractive-index materials, PCs enable precise control over light–matter interactions, facilitating the development of low-loss optical waveguides and high-speed photonic integrated circuits that underpin modern telecommunication technologies.^1^

One-dimensional defective photonic crystals (1D-DPCs) are optical systems formed by a periodic modulation of refractive indices along a single axis, where the intentional introduction of a defect layer breaks the periodic order. This defect gives rise to a localized transmission resonance within the photonic bandgap (PBG), whose spectral position is highly sensitive to variations in the refractive index of the defect region.^2^ Owing to this property, 1D-PCs have been widely employed as sensing platforms in environmental, biomedical, and industrial applications.^3,4^ Extensive research has focused on enhancing the sensing performance of 1D-DPC-based devices by optimizing key parameters such as sensitivity, quality factor (*Q*), and figure of merit (FOM).^5–7^ Notably, highly efficient sensors have been reported for waterborne bacteria detection, achieving sensitivities as high as 2896.82 nm RIU^−1^, *Q*-factors exceeding 4243, and FOM values reaching 4298.58 RIU^−1^.^8^

Phase-change materials (PCMs) are a class of functional materials that can undergo reversible transitions between amorphous and crystalline phases when stimulated thermally or optically, leading to pronounced variations in their optical and electrical characteristics.^9,10^ Within photonic crystal systems, PCMs—most notably germanium antimony telluride (GST)—are incorporated to actively control the photonic bandgap through phase-dependent refractive-index modulation, allowing the realization of reconfigurable photonic components such as optical filters and biosensors. This controllable behavior is especially advantageous in one-dimensional defective photonic crystals (1D-DPCs), where embedding PCMs in the defect region enables precise tuning of the defect-mode resonance wavelength, thereby improving sensing performance. Previous studies have confirmed that photonic crystals integrated with PCMs exhibit enhanced quality factors and high sensitivity, making them highly suitable for advanced biosensing applications.^11,12^

GST in photonic crystals is highly important due to its reversible phase transition between amorphous and crystalline states under thermal or optical stimulation, enabling dynamic modulation of the photonic bandgap.^13,14^ This transition causes significant changes in refractive index and reflectivity, allowing precise tuning of the defect mode for reconfigurable devices like filters and sensors. GST also offers high optical and electrical contrast between phases, enhancing sensitivity to environmental changes, such as biological interactions, and improving *Q*-factor and detection capability for high-precision biosensing.^15,16^

GST plays a significant role in photonic devices due to its reversible phase transition between amorphous and crystalline states under thermal, optical, or electrical stimuli. This switching causes large changes in refractive index, enabling reconfigurable and tunable devices with high contrast, fast switching speed, thermal stability, and compatibility with thin-film fabrication.^17–26^ GST allows active tuning of photonic structures by altering their spectrum through phase transitions, making it ideal for dynamic optical control. The high refractive index contrast enhances sensitivity and performance in biosensing applications, such as label-free detection of biological samples (*e.g.*, reproductive hormones).^21–26^

A promising use for PCs is in biosensing, where their light enhancement and environmental sensitivity help in the detection of biomolecular interactions. As an example, PC-based biosensors can measure a change in refractive index due to biomolecules like proteins or DNA binding to the sensor surface. PC biosensors measure the binding of target biomolecules by measuring the refractive index of the defect layers made with biomolecules like proteins, DNA, or even pathogens that change the resonance wavelength.^27–30^

The thyroid gland, a butterfly-shaped organ found at the front of the neck, is responsible for fueling a multitude of physiological functions critical to maintaining good health. The gland produces two primary hormones, thyroxine (T4) and triiodothyronine (T3), which are essential for controlling the body's metabolism, that is, the rate at which food is converted to fuel for cellular activities. Almost all body tissues are impacted by these hormones, which help to modulate the basic metabolic activities, oxygen intake, and energy use in the body.^31^ Thyroid hormones are also important beyond metabolism, especially during the early phases to foster growth and development.^32^ It manages linear growth, brain development, neural function, intelligence, memory, dentition, and bones. If congenital hypothyroidism is not detected and treated timely manner, it will result in irreversible brain damage and profound mental disability.^33^

The thyroid gland also secretes calcitonin, which helps maintain calcium balance in the blood by promoting calcium deposition in bone (and hence bone health).^34^ The thyroid also impacts cardiovascular functioning, resting heart rate, mood, energy, and reproductive health, so its normal functioning is crucial for health, and its dysfunctions can contribute to systemic symptoms such as weight fluctuations, fatigue, temperature sensitivity, and even hyperthyroid cardiomyopathy or hepatic injury in severe hyperthyroidism.^35^ The gland also has a function in controlling the immune response, and is involved in autoimmune thyroid diseases (including Hashimoto's thyroiditis).

Several techniques are presently employed for diagnosing thyroid nodules, including ultrasonography, fine-needle aspiration cytology (FNAC), and biochemical testing. Despite their widespread use, these methods may suffer from limitations such as operator dependence, invasiveness, and reduced sensitivity when distinguishing early-stage benign from malignant nodules. In this regard, terahertz time-domain spectroscopy (THz-TDS) has emerged as an attractive alternative owing to its intrinsic advantages. THz radiation is non-ionizing and biologically safe, with photon energies in the millielectronvolt range that do not induce molecular damage, in contrast to conventional X-ray-based techniques.^36,37^ Moreover, THz-TDS enables non-contact, label-free analysis without the need for contrast agents or chemical markers, thereby allowing direct interrogation of native biomolecular characteristics.^38,39^

Importantly, THz-TDS is highly responsive to collective molecular dynamics, intermolecular interactions, and hydration levels—parameters that undergo significant modification in malignant tissues as a result of changes in protein structure, metabolic activity, and water content.^40^ The technique also permits concurrent extraction of absorption and refractive-index spectra, offering insight into subtle dielectric variations in blood plasma that mirror underlying biochemical alterations associated with thyroid cancer.^41,42^ Previous studies demonstrating successful discrimination between benign and malignant states in breast, skin (including basal cell carcinoma), colorectal, and gastric cancers further highlight the diagnostic potential of THz-TDS.^43,44^ Consequently, THz-TDS represents a promising complementary tool for thyroid nodule assessment, enabling rapid, non-invasive plasma-based screening and the identification of disease-specific spectral signatures linked to biomarkers such as glucose and microRNAs.^45^

The early diagnosis of thyroid gland pathology has become urgent due to its implications for global health and relations with other systemic diseases. Recent studies emphasize the importance of early intervention to avert grave consequences and enhance life quality. For example, a study highlights recent advancements in thyroid function testing that enable more accurate diagnostics and support precision medicine with more personalized treatment approaches. The same study, however, argues that poor diagnosis can have far-reaching impacts that do not optimally serve the patient's interests, particularly in subclinical hypothyroid patients, who, if unnoticed, may progress into overt disease.^46^ This is critical because the most common endocrine malignancy—thyroid cancer—necessitates early diagnosis to improve the prognosis due to its rising incidence worldwide.^47^

Most recently, other areas of concern have come to light that highlight thyroid dysfunction and its relation to other health conditions, which raises the most concern around screening in high-risk groups. Studies show that thyroid dysfunction—particularly hypothyroidism—plays a role in the development of metabolic syndrome, which is linked to cardiovascular diseases and type 2 diabetes.

As reported in ref. 48, the study advised routine thyroid screening for women—especially those who are postmenopausal—with metabolic abnormalities, as they were previously shown to have a higher prevalence. Similarly, a study on patients with COVID-19 and biochemically confirmed thyroid dysfunction found higher rates of complications and mortality, highlighting the importance of early identification and effective management of thyroid disorders during pandemics to reduce adverse outcomes.

In this study, a key novelty of the proposed approach is the systematic comparison of THz-TDS measurements on both liquid and lyophilized plasma. Revealing that the lyophilized pellet format provides substantially improved discrimination between malignant and benign thyroid nodules due to the removal of bulk water interference, thereby exposing subtle dielectric variations associated with biomolecular changes. The 1D-DPC structure is designed as a biosensor for the detection of thyroid cells. Each time a defective layer in 1D-PC is refilled with healthy thyroid tissue and thyroid nodules. These nodular cells have different refractive indices, consequently affecting the appearance and position of a resonant peak in the PBG. The 1D-DPC is very sensitive to the change in the refractive index of the small defect layer due to the strong confinement of the incident electric field.

## 2 Theory

**Fig. 1:**
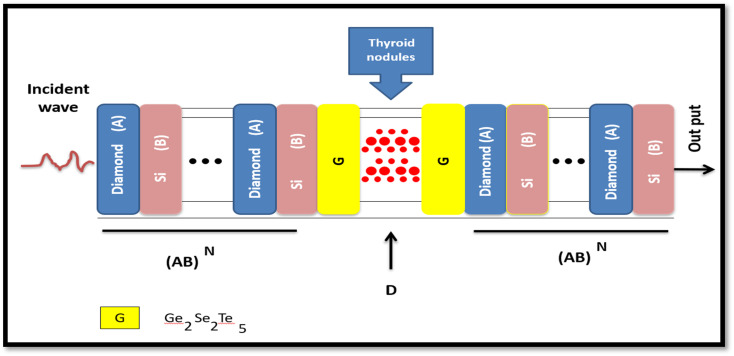
The schematic diagram of a 1D-DPCs biosensor [(Diamond/Silica)^3^ GDG (Diamond/Silica)^3^].

The proposed 1D-DPC biosensor (AB)^N^GDG(AB)^N^ is shown in Fig. 1. A is referred to Diamond layer, B is the porous silicon layer, G is the phase change material (PCM) which is called Ge_2_Sb_2_Te_5_ (GST), and finally D is the defect layer. The defect layer D, whose both sides are coated with two identical buffer layers (GST). The defective layer is blood plasma from healthy individuals and patients with thyroid gland nodules. The whole structure is surrounded by a SiO_2_ substrate on one side and air on the other side.

The constituent dielectric materials of the designed 1D-PC are the first layer, Diamond layer with thickness *d*_1_ = 62 nm and refractive index *n*_1_ = 2.4168, and the second layer, porous silicon (PS) layer with thickness *d*_2_ = 125 nm and refractive index *n*_2_ = 1.2.^49–51^ The thickness of layer G (GST) *d*_G_ = 40 nm, and the complex refractive index values of this material under amorphous and crystalline phases have been used to give real and imaginary values of GST material under amorphous and crystalline phases.^16^

### 2.1 The refractive index of porous silicon (PS)

Porous silicon (PS) has emerged as a cornerstone material in photonic crystal (PC) engineering due to its tunable refractive index, facile fabrication *via* electrochemical anodization, and compatibility with silicon-based technologies, enabling high-contrast multilayer structures for applications in optical sensors, Bragg reflectors, and Tamm plasmon devices. Specifically, a low refractive index layer with *n* = 1.2—achieved at high porosity of 65–68% using heavily doped p-type silicon (*ρ* = 0.005–0.01 Ω cm^−1^, (100) orientation), current density *J*_L_ = 10–18 mA cm^−2^, etching time *t*_L_ = 15–30 s, and HF: ethanol electrolyte (1 : 1 or 3 : 7 ratio)—serves as the critical low-index (L) layer in 1D PC designs to maximize photonic bandgap width and sensing sensitivity (>100 nm RIU^−1^). This effective index is reliably calculated *via* the Bruggeman effective medium approximation and validated experimentally through spectroscopic ellipsometry and reflectance fitting. Key studies demonstrating this exact configuration include, *ACS Photonics* 2020, reporting *n*_L_ = 1.2 at *J*_L_ = 15 mA cm^−2^ and 66% porosity in multilayer sensors, Optics Express 2018, confirming *n* = 1.2 with porosity 65% in optical microcavities, and *Sensors and Actuators B: Chemical* 2021, utilizing *n* = 1.2 (porosity 68%, *J*_L_ = 12 mA cm^−2^) for biomolecule detection.^49–51^ These references provide reproducible protocols and empirical validation for integrating *n* = 1.2 PS layers in photonic architectures.

### 2.2 The refractive index of the GST material

GST thin films are typically fabricated by RF magnetron sputtering at room temperature to obtain the amorphous phase, with thickness controlled to 50–200 nm.^52,53^ Crystallization is induced by annealing at 140–180 °C in inert atmosphere. For biosensing compatibility, reversible phase switching is achieved non-thermally (*e.g.*, femtosecond laser pulses for amorphization and near-infrared illumination for crystallization) or with low-power electrical heating to avoid thermal damage to biological samples.^54,55^ This ensures significant optical contrast while preserving analyte integrity. The overall multilayer photonic crystal can be realized using standard thin-film deposition techniques (*e.g.*, sputtering or evaporation for Diamond/Silica layers) and sacrificial etching for the defect cavity, as demonstrated in similar GST-based structures.

The following equations give theoretical values of complex refractive index of GST material under amorphous and crystalline phases used in this study, respectively^14,16^

1 *N*_A_ = *n*_AGST_ + *iK*_AGST_

2 *N*_C_ = *n*_CGST_ + *iK*_CGST_

In the amorphous phase *n*_AGST_ and *K*_AGST_ are representing the real and imaginary parts of the complex refractive index of GST.

3 *n*_AGST_ = −0.51929*λ*^4^ + 4.3531*λ*^3^ − 12.984*λ*^2^ + 15.789*λ* − 2.0315

4 *K*_AGST_ = −0.29346*λ*^3^ + 2.3145*λ*^2^ − 5.9547*λ* + 4.9925

Also, in the crystalline phase *n*_CGST_ and *K*_CGST_ are representing the real and imaginary parts of the complex refractive index of GST.

5 *n*_CGST_ = 1.2751*λ*^3^ − 8.1973*λ*^2^ + 16.168*λ* − 2.9464

6 *K*_CGST_ = −0.72443*λ*^4^ + 5.461*λ*^3^ − 13.33*λ*^2^ + 9.7612*λ* + 1.8907

GST permittivity is dispersive in the visible range, with both real (*ε*′) and imaginary (*ε*″) parts varying with wavelength. The imaginary part (*ε*″ = 2*nk*) accounts for absorption, causing moderate resonance broadening and slight *Q*-factor reduction compared to lossless models. However, in the selected operating window, absorption remains low, maintaining high *Q*-factors (>80) and sharp resonances suitable for biosensing. This dispersive model ensures realistic performance predictions.^52,53^

### 2.3 The refractive indices of the samples that filled the defect layer

The refractive index of healthy individuals and patients with thyroid nodules has been theoretically calculated by the following equations.

7 *n*_water_ = 5.0063 × 10^−7^ × *λ* + 2.2388

8 *n*_Thyroid nodules_ = 4.5184 × 10^−7^ × *λ* + 2.1910

9 *n*_Healthy_ = 1.6224 × 10^−7^ × *λ* + 2.0199

The refractive indices of malignant thyroid nodules without water and benign thyroid nodules without water as a function of wavelength are calculated by the following equations.

10 *n*(*λ*)_malignant nodules without water_ = −1.062390 × 10^−13^ × *λ*^2^ − 2.452184 × 10^−7^ × *λ* + 9.657855 × 10^−1^

11 *n*(*λ*)_benign nodules without water_ = 1.743917 × 10^−13^ × *λ*^2^ − 3.124736 × 10^−7^ × *λ* + 9.921420 × 10^−1^

These eqn (7)–(11) were derived from dielectric relaxation models applied to THz-TDS measurements of lyophilized plasma, as detailed in Section 3.1 (Results, page 10) and based on the published data in Konnikova *et al*.^56^

### 2.4 Transfers matrix method (TMM)

Here, the same constituent dielectric materials are used, with a defective layer considered for thyroid nodules with different refractive indices. When electromagnetic waves (IR-waves) incident on the photonic crystal, some photons can propagate through the crystal, and other photons are expected to be totally reflected. Thus, crystal acts as a high reflectance reflector for the range of incident frequencies.^42^ The forbidden photons with certain frequencies lead to the appearance of the photonic band gap (PBG). To realize the PBG effect in transmission spectra, the TMM was used. In this method, each layer has its own matrix specifying the dielectric parameters, and the total transfer matrix of the whole structure is calculated by multiplying all the single-layer transfer matrices.^57–59^ The transfer matrix for each layer is given by12
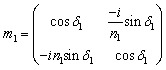
where l represents the Diamond layer, the Silica layer either or the Thyroid nodules layer.

The phase *δ*_1_ is expressed as:13
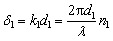
where *n*_l_, *d*_l_ and *k*_l_ are the refractive index, the thickness, and the wave vector of the structure layers.

The single-period matrix is shown in the following equations.14
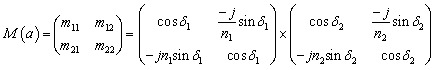


Since *a* = (*d*_1_ + *d*_2_) is the lattice constant. For the whole structure of N-periods, the total characteristic matrix *M* can be obtained using the same expressions that are used in eqn (1) and (2) for the total transfer matrix is given by:

15 *M* = (*M*_A_*M*_B_)^N^(*M*_GDG_)(*M*_A_*M*_B_)^N^

A, B, and D represent the Diamond, Si, defective layer (PCM/thyroid cell/PCM), respectively, and N is the N-periodic layers.

Finally, the transmittance of the whole structure is given by:16
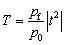
where;17
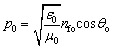
18
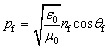
And *t* is the transmission coefficient which is given by19
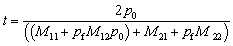


### 2.5 The performance parameters

The evaluation of the performance and efficiency of the proposed design has been done by calculating sensitivity (*S*), quality factor (*Q*), and figure of merit (FOM) values because these are the most common parameters to evaluate the performance of any biosensor. The sensitivity is the ratio of the change in the central wavelength of the defect mode (Δ*λ*) due to a change in the refractive index of the cavity region (Δ*n*) as defined below.^27^20
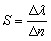
where Δ*λ* is the wavelength shift of peak resonance caused by the change of Δ*n*. By considering the wavelength of the resonant peak and RI of the normal cell as a reference, we found that Δ*λ* = (*λ*_Healthy_ − *λ*_Thyroid nodules_)/Δ*n* (*n*_Healthy_ − *n*_Thyroid nodules_).^28^

For getting accuracy in biosensor measurements, the quality factor (*Q*) of the biosensor should be as high as possible, and it can be calculated with the help of the following equation.21
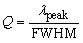
where *λ*_peak_ and FWHM are used to represent the central wavelength and full width at half maximum of the transmission peak inside the cavity region.

The figure of merit (FOM) of biosensor design is defined as the ratio of sensitivity to FWHM of the defect mode inside PBG. FOM can be obtained by using the following relation.22
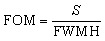


## 3 Results

### 3.1 The fitting experimental data of the refractive indices of the samples that filled the defect layer

**Fig. 2:**
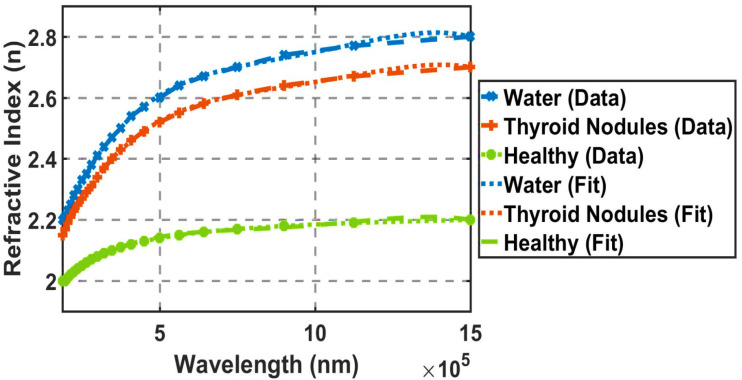
Refractive indices of water, thyroid nodules, and healthy thyroid as a function of wavelength (nm). These values have been obtained by applying Engauge Digitizer on published experimental data.^56^

Fig. 2 illustrates the refractive indices of water, blood plasma from healthy individuals and patients with thyroid gland nodules with the wavelength. These results were derived from an experimental study,^60^ where the Digitizer Engauge program was used to extract experimental values as numerical data. These data were plotted to confirm the same behavior Fig. 2. The equations describing these relationships between wavelength and refractive index were calculated based on eqn (7)–(9). This figure illustrates the preliminary diagnosis of the healthy and nodular conditions of the thyroid, referred to as the “first stage” in the practical study.

A pronounced inverse relationship between glucose concentration and the terahertz (THz) absorption coefficient has been observed in lyophilized plasma samples obtained from patients with thyroid nodules.^40^ In the THz spectral domain, the dielectric response of blood plasma is extremely sensitive to its complex biochemical makeup, which consists of roughly 92% water, 7–8% proteins, electrolytes, glucose, lipids, and trace-level biomolecules such as microRNAs.^60^ THz radiation couples strongly to intermolecular hydrogen-bond networks and collective molecular vibrations, rendering the complex dielectric function, *ε*(*ω*) = *ε*′(*ω*) − *iε*″(*ω*), highly responsive to variations in hydration state, protein structure, and metabolite content.^40,59^ In liquid plasma, water overwhelmingly governs the dielectric behavior due to its intense Debye relaxation and characteristic absorption features in the 0.5–1 THz range, thereby obscuring weaker signals arising from other constituents.^39,60^ Lyophilization effectively eliminates bulk water, substantially suppressing this dominant absorption and unveiling the dielectric contributions of proteins, glucose, and nucleic acids. Variations in glucose concentration, for example, influence local polarization mechanisms and hydrogen-bond dynamics, producing detectable changes in both the refractive index and absorption coefficient.^55^ Likewise, alterations in protein secondary structure or elevated microRNA expression associated with malignant conditions can perturb low-frequency collective modes that are accessible in the THz regime.^43^ These composition-dependent dielectric effects underpin the spectral distinctions observed between benign and malignant thyroid nodule plasma in the present study. Moreover, this contrast is intensified in malignant samples as a consequence of the Warburg effect, whereby enhanced glucose uptake by cancer cells lowers plasma glucose levels, leading to increased THz absorption in lyophilized plasma pellets.^40,59^

**Fig. 3:**
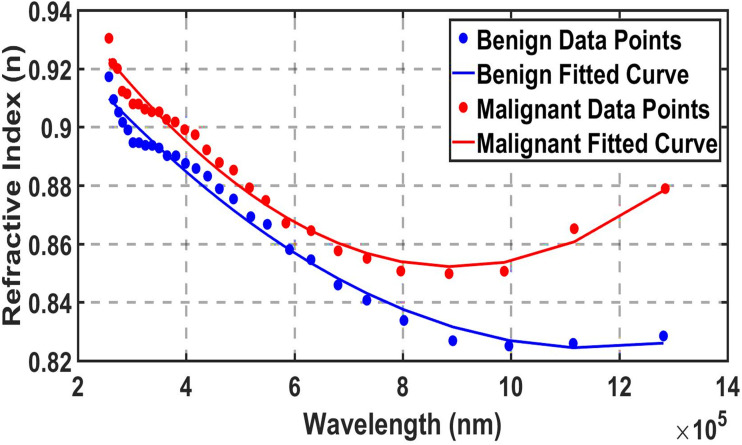
Refractive indices of malignant thyroid nodules and benign thyroid nodules without water as a function of wavelength (nm). These values were obtained by applying Engauge Digitizer to published experimental data.^56^

Subsequently, differentiation is made between benign and malignant nodular conditions, as described in the practical study^56^ as the “second stage”. This stage is shown in Fig. 3, which was created using the same method as Fig. 2, utilizing the Engauge Digitizer program. However, in this case, the effect of water in the sample was removed, resulting in the refractive index of benign and malignant nodules ranging between 0.9 and 0.95. This technique is called the spectral subtraction technique, defined as *n*_dif_ = *n*_plasma_ − *n*_water_, serves as a cornerstone differential method for isolating disease-specific biomolecular refractive index signatures in blood plasma by eliminating the dominant water background, thereby amplifying subtle pathological variations with enhanced signal-to-noise ratio and specificity. This approach has been extensively validated in reviewed biosensing studies, enabling label-free cancer diagnostics across multiple modalities; for example, it facilitated precise differentiation of malignant *versus* benign thyroid nodules with over 90% accuracy through terahertz plasma analysis.^56^ In our study, an initial screening to distinguish healthy from thyroid nodules, but a more refined diagnosis was required to accurately classify nodule type (benign or malignant); thus, we employed spectral subtraction to extract as *n*_dif_ the sensing parameter within our design. This methodology extends robustly to other cancers, including breast,^61^ colorectal,^62^ and pancreatic,^63^ establishing spectral subtraction as a versatile, non-invasive tool for multiplexed liquid biopsy when coupled with photonic architectures.

Fig. 2 and 3 demonstrate sufficient agreement with the experimental results.^56^ This indicates that the fitting equations are suitable for determining the refractive indices of the samples that filled the defect layer, and that the fitting procedure yields results consistent with the experimental data. The equations describing Fig. 3 are also shown in eqn (21) and (22). As the concentration increases, the refractive index also increases.^26^ Consequently, the refractive index for the thyroid nodules is higher than that for the healthy thyroid, as shown in Fig. 2. Similarly, the refractive index for malignant nodules is higher than that for benign nodules, as illustrated in Fig. 3.

The spectral contrast between benign and malignant liquid plasma samples was notably weaker compared to lyophilized pellets. This observation is primarily attributed to the dominant broadband absorption of water, which constitutes approximately 92% of blood plasma volume. Water exhibits strong Debye relaxation and resonant absorption in the THz range (peaking around 0.5–1 THz and extending broadly up to 2 THz), resulting in high attenuation coefficients that overwhelm the subtle dielectric contributions from biomolecules such as proteins, glucose, and miRNAs.^36,56^ Consequently, the intense water absorption masks the low-amplitude spectral features arising from biochemical differences between benign and malignant samples.

In contrast, lyophilization removes bulk water, exposing these subtler contributions and enabling clearer correlations with specific biomarkers. For instance, the reported positive correlation between miRNA-146b levels and THz absorption in dry plasma^56^ can be linked to the overexpression of miRNA-146b in malignant thyroid nodules, which modulates inflammatory pathways and promotes oncogenesis.^64^ Higher miRNA-146b may induce conformational changes in circulating nucleic acids and associated proteins, enhancing low-frequency collective vibrational modes and intermolecular interactions detectable in the THz range.^55^ Similarly, alterations in glucose levels (influenced by the Warburg effect in malignant cells) contribute to the observed negative correlation with absorption in lyophilized samples.

Malignant lyophilized plasma samples show higher THz absorption than benign ones. This is due to the Warburg effect: cancerous cells consume more glucose, lowering plasma glucose levels and weakening hydrogen-bonding networks. The result is more free collective vibrational modes and an increased imaginary part of the dielectric function, leading to stronger broadband THz absorption.^55,65^ This effect is further amplified in malignant samples due to the Warburg effect, where cancerous cells exhibit increased glucose uptake, reducing plasma glucose levels and resulting in higher THz absorption in lyophilized pellets due to altered hydrogen-bonding dynamics.^40,64^ Similarly, the positive correlation with miRNA-146b levels^56^ reflects its overexpression in malignant thyroid nodules.^59^ Elevated miRNA-146b may induce conformational changes in circulating nucleic acids, enhancing low-frequency collective vibrational modes detectable in the THz range.^66^ These biomarker correlations provide a biophysical basis for the enhanced spectral separation observed in lyophilized plasma (Fig. 2 and 3), supporting THz-TDS as a promising tool for reflecting metabolic and molecular alterations in thyroid pathologies.

### 3.2 Clinical applicability prospects of THz-based plasma screening in a theoretical framework with photonic biosensor integration

Terahertz time-domain spectroscopy (THz-TDS) analysis of blood plasma offers a promising non-invasive, label-free method for differentiating benign from malignant thyroid nodules. Current clinical practice relies primarily on ultrasound imaging followed by fine-needle aspiration cytology (FNAC), which is invasive and yields indeterminate results in 15–30% of cases, often leading to unnecessary diagnostic surgeries.^53,67^ In contrast, THz-based plasma screening requires only a routine blood draw, followed by lyophilization and rapid spectroscopic measurement, potentially serving as a complementary tool to stratify nodule risk and reduce the need for invasive procedures.

The dielectric signatures obtained from THz plasma screening can be directly translated into advanced photonic biosensor platforms. In particular, the distinct variations in absorption coefficient and refractive index between malignant and benign plasma samples can guide the design of one-dimensional defect photonic crystal (1D-DPC) structures incorporating phase-change materials such as Ge_2_Sb_2_Te_5_ (GST). By introducing a single defect layer functionalized for plasma-derived biomolecules, the PhC resonance in the THz range becomes highly sensitive to subtle refractive index changes induced by malignant samples, enabling defect-mode shifting for enhanced detection specificity.^68^ This integration paves the way for compact, reusable THz photonic biosensors suitable for point-of-care thyroid disease diagnostics, combining the rich spectral information from bulk plasma analysis with the high sensitivity and miniaturization of defect-based photonic sensing.

THz-TDS plasma screening can complement existing tools such as ultrasound-guided FNAC and biochemical assays. FNAC is invasive and yields indeterminate results in 15–30% of cases, often requiring repeat procedures or surgery.^67,69^ Biochemical assays lack specificity for malignancy in indeterminate nodules. In contrast, THz-TDS is non-invasive (routine blood draw only), rapid, and provides biophysical insights into metabolic/molecular changes, potentially improving risk stratification and reducing unnecessary invasive interventions when used adjunctively.

The present study is a theoretical simulation-based investigation that utilizes published experimental THz-TDS data from blood plasma samples,^60^ with no new experimental measurements performed. Accordingly, the sample size is limited to that of the original study, restricting statistical power and generalizability. The results depend on the characteristics of the published dataset, and external experimental validation on independent, larger cohorts is required to confirm the simulated performance of the proposed one-defected photonic crystal biosensor. Future work should include practical fabrication and testing with fresh plasma samples to translate the theoretical findings into clinical application.

### 3.3 The transmission spectra of the “first stage” under amorphous and crystalline phases of GST material

**Fig. 4:**
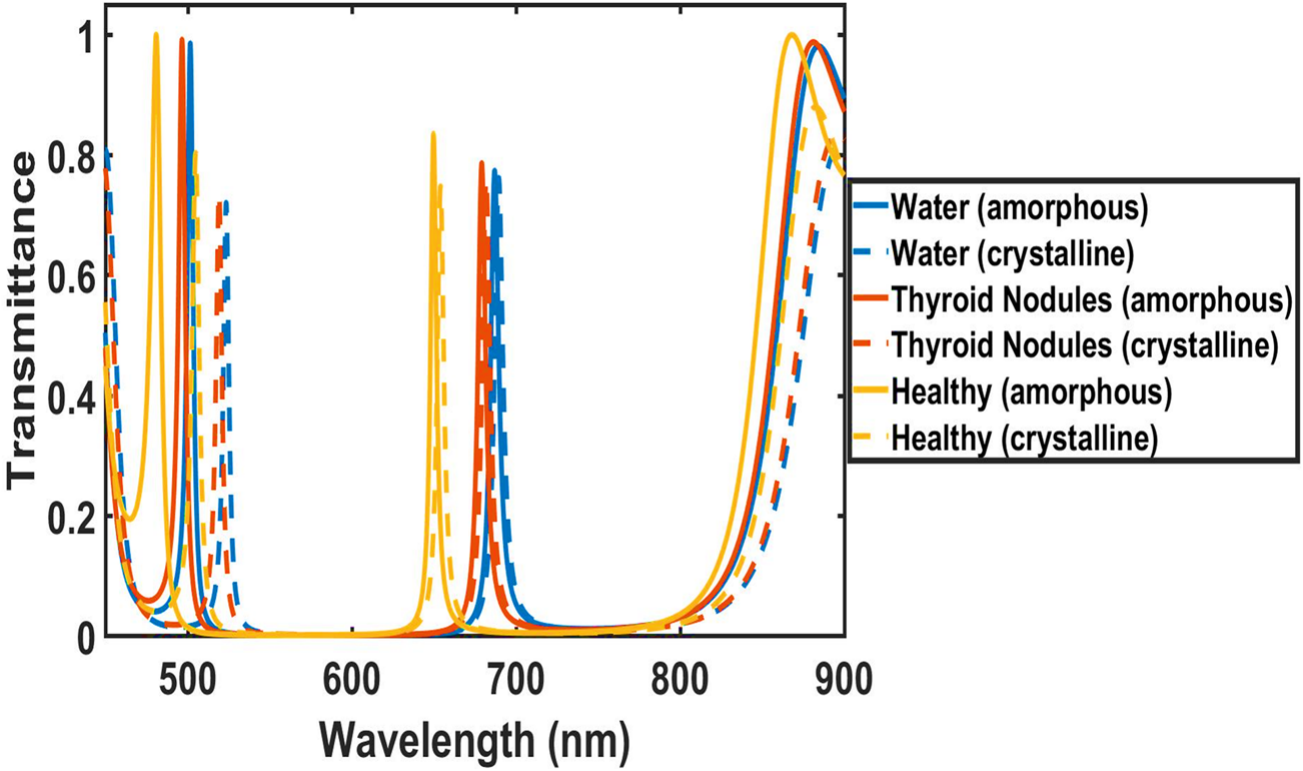
Transmission spectra of the biosensor [Diamond/Silica)^3^ GDG (Diamond/Silica)^3^] at normal incidence loaded with different samples one by one with cavity of thickness dd = 220 nm under crystalline phase and amorphous.

Fig. 4 shows the transmission spectra of the (1D-DPC) biosensor [Diamond/Silica)^3^ GDG (Diamond/Silica)^3^] at normal incidence, under the influence of amorphous and crystalline phases of GST material in the case of the first stage. The TMM was applied using MATLAB software to obtain the results of this study. The structural parameters of the proposed biosensor are defined in the previous section. To carry out the investigations of the first stage, water, thyroid nodules, and a healthy thyroid were poured one by one into the defect layer region D, surrounded by PCM (Ge_2_se_2_Te_2_), across the wavelength range of 450–900 nm.

As shown in Fig. 4, the bandgap shifts to longer wavelengths (redshift) when the PCM transitions from the amorphous to the crystalline state. For water, the bandgap moves from approximately 513 nm (amorphous) to 535 nm (crystalline). For healthy thyroid tissue, it shifts from around 500 nm (amorphous) to 518 nm (crystalline), and for thyroid nodule tissue, it changes from about 511 nm (amorphous) to 529 nm (crystalline). This redshift is due to the increased refractive index in the crystalline state, which alters the effective optical path length and widens the bandgap, consistent with the material's phase-dependent properties.

Table 1 shows the performance parameters of our design in the first stage, in both amorphous and crystalline states of the phase change material. The defect modes exhibit wavelength shifts (Δ*λ*) that serve as a primary signature for distinguishing water, healthy thyroid cells, and thyroid nodules in both amorphous and crystalline phases. In the crystalline phase the mode red-shifts to 654.1 nm (healthy), 681.7 nm (nodules), and 689.5 nm (water). The amorphous state amplifies this separation, the defect mode shifts from 650 nm (healthy) to 679 nm (nodules) to 687 nm (water).

**Table 1:** The performance parameters of our design in the first stage

Material	State	Peak wavelength (nm)	Refractive index (RIU)	Sensitivity (nm RIU^−1^)	*λ*_FWHM_ (nm)	*Q*	FOM (1/RIU)
Water	Amorphous	687	2.23914	—	6.10	112.6229	—
	Crystalline	689.5	2.2391	—	6	114.9166	—
Thyroid nodules	Amorphous	679	2.1913	167	5.64	120.3900	29.6496
	Crystalline	681.7	2.1913	163	6	113.6166	27.1966
Healthy	Amorphous	650	2.0200	168	4.3	151.1627	39.2655
	Crystalline	654.1	2.0200	161	4.9	133.4897	32.9734

The photonic crystal sensor exhibits consistently sensitivity, ranging from 161 to 168 nm RIU^−1^, with the highest sensitivity of 168 nm RIU^−1^ achieved in healthy tissue under the amorphous phase—surpassing thyroid nodules (167 nm RIU^−1^ amorphous, 163 nm RIU^−1^ crystalline) and healthy crystalline (161 nm RIU^−1^). This same configuration—healthy tissue in amorphous state—also delivers the highest *Q*-factor of 151.16, significantly outperforming all other conditions (*e.g.*, 133.49 in healthy crystalline, 120.39 in nodular amorphous). Overall, the biosensor displays enhanced detection capabilities for thyroid nodules and healthy tissue compared to water, with the amorphous state generally yielding better metrics across all parameters, suggesting its preference for optimal biosensor performance in biomedical applications.

**Fig. 5:**
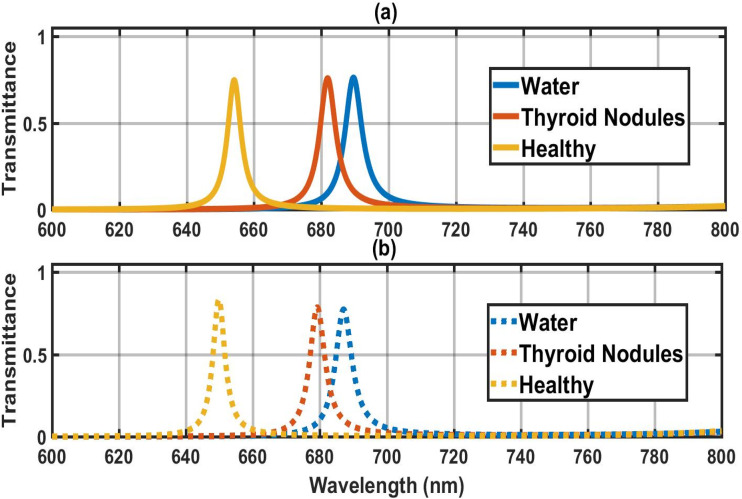
Transmission spectra of defect modes of the biosensor [Diamond/Silica)^3^ GDG (Diamond/Silica)^3^] at normal incidence loaded with different samples one by one with cavity of thickness dd = 220 nm (a) under crystal and (b) under the amorphous.

Fig. 5a depicts that under the crystalline phase, the three distinguishable defect modes corresponding to water, Thyroid nodules, and healthy thyroid cells are found at 689 nm, 681 nm, and 654 nm inside the PBG, respectively. The reason for these distinguishable defect modes in the side PBG is due to a minute change in effective refractive indices as described in eqn (1)–(3). The standing wave concept of a laser cavity can also be used to understand the presence of a defect mode inside the PBG. According to the standing wave concept of a laser cavity, only those wavelengths inside the cavity can come out that satisfy the relation *δ* = *k λ* = *n*_eff_*L*. Here, notations *δ*, *k*, *λ*, *n*_eff,_ and *L* have been used to represent optical path difference, an integer, wavelength inside cavity, effective refractive index, and cavity length, respectively. The transformation of GST from AGST to CGST phases under the above-mentioned circumstances exhibits a significant change in defective mode location inside PBG. Fig. 5b shows that under the amorphous phase of GST material, defect modes of different cases, water, Thyroid nodules, and health have repositioned their location at 687 nm, 679 nm, and 649 nm.

### 3.4 The transmission spectra of the “second stage” under amorphous and crystalline phases of GST material

**Fig. 6:**
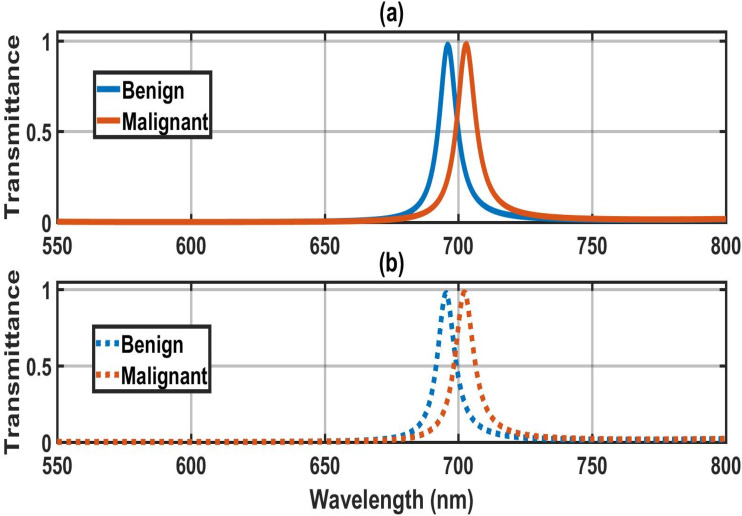
Transmission spectra of defect modes of the biosensor [Diamond/Silica)^3^ GDG (Diamond/Silica)^3^] at normal incidence loaded with malignant thyroid nodules without water and benign thyroid nodules without water, one by one, with cavity (a) under crystalline and (b) under the amorphous.

The transmission spectra of defect modes of the (1D-DPC) biosensor [(Diamond/Silica)^3^ GDG (Diamond/Silica)^3^] under amorphous and crystalline phases of GST material corresponding to malignant and benign thyroid nodules without water (second stage) are plotted in Fig. 6a and b, respectively. Fig. 6a depicts that under crystalline phase GST material, the two defect modes corresponding to malignant and benign thyroid nodules without water are found at 703 nm and 696 nm inside the PBG, respectively. Fig. 6b shows that under the amorphous phase of GST material, defect modes of malignant and benign thyroid nodules without water have repositioned their location at 702 nm and 695 nm, respectively. The defect mode of benign thyroid cells is blue-shifted from the defect mode of malignant thyroid cells in all phases of GST material.

(1D-DPC) biosensor design successfully demonstrates a clear distinction between the first and second stages of thyroid nodule disease. The design's sensitivity to structural modifications is demonstrated by its ability to distinguish between the two phases, which makes it an effective and accurate method for identifying minute optical changes. As a result, the suggested 1D-DPC may be regarded as a possible diagnostic method for the identification of thyroid gland disorders.

Table 2 shows the photonic crystal sensor effectively distinguishes benign from malignant thyroid nodules in the absence of water through pronounced spectral shifts and performance metrics. In the amorphous state, the defect mode resonates at 695.5 nm for benign nodules and red-shifts to 702.0 nm for malignant ones. Sensitivity of malignant cases, reaching 245 nm RIU^−1^ for amorphous and a higher 267 nm RIU^−1^ for crystalline, indicating stronger wavelength response to refractive index changes in the crystalline configuration. Both states show broader resonance (FWHM = 8–8.5 nm). Thus, while the crystalline phase shows superior sensitivity and FOM for malignant detection, the red-shift across phases enables label-free differentiation of nodule malignancy primarily *via* peak wavelength position.

**Table 2:** The performance parameters of our design in the second stage

Material	State	Peak wavelength (nm)	Refractive index (RIU)	Sensitivity (nm RIU^−1^)	*λ*_FWHM_ (nm)	*Q*	FOM (1/RIU)
Benign without water	Amorphous	695.50	0.9921	—	8	86.9375	—
	Crystalline	696.00	0.9919	—	8	87.000	—
Malignant without water	Amorphous	702.00	0.9656	245	8.5	82.5882	28.8568
	Crystalline	703.00	0.9657	267	8	87.875	33.3969

### 3.5 Effect of the thickness of the defect layer on the transmission spectra of the “first stage”, under amorphous and crystalline phases of GST material

In this section, we have estimated the performance of the proposed photonic biosensor by changing the thickness of the cavity region from 230 nm to 245 nm when the cavity is loaded with healthy thyroid and thyroid nodules under crystalline and amorphous phases of GST material.

**Fig. 7:**
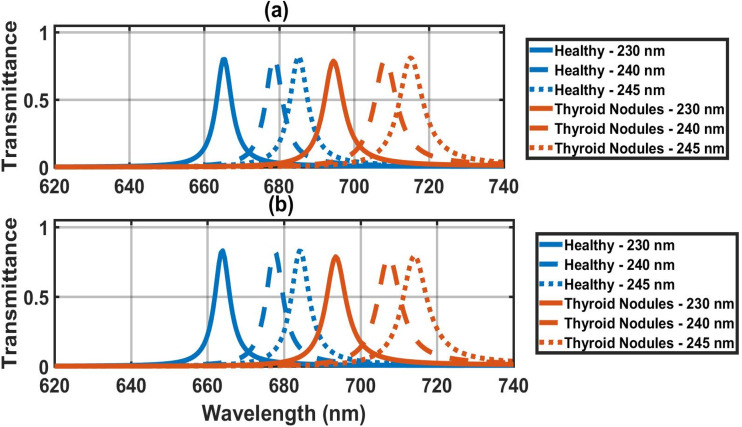
Transmission spectra of defect modes of the (1D-DPC) biosensor [Diamond/Silica)^3^ GDG (Diamond/Silica)^3^] loaded with healthy thyroid and thyroid nodules at *θ* = 0° and different defect layer thicknesses dd = 230 nm, 240 nm, 245 nm (a) under crystalline phase of GST and (b) under amorphous phase of GST.

Transmission spectra of the proposed biosensing design at normal incidence angle with defect layer thicknesses 230 nm, 240 nm, and 245 nm under the influence of crystalline and amorphous phases of GST material have been plotted in Fig. 7a and b, respectively. As the thickness of the cavity region increases from 230 nm to 245 nm, the position of the defect mode inside PBG evenly shifts toward the higher wavelength side between 664 nm and 720 nm inside PBG under the amorphous phase of GST. And the defect modes are red-shifted from 665 nm to 715 nm under the crystalline phase of GST.

Table 3 shows the performance parameters of our design with different defect thicknesses (220 nm, 230 nm, 240 nm, 245 nm) at crystalline phase of GST for the first stage. The photonic crystal sensor operating in the crystalline GST phase exhibits a systematic thickness-dependent red-shift of the defect mode for both healthy thyroid tissue and nodules, enabling tunable spectral discrimination. For healthy tissue increasing defect-layer thickness from 220 nm to 245 nm shifts the resonance from 654.1 nm to 685 nm, with FWHM widening from 4.9 nm to 5.5 nm beyond 230 nm. In contrast, thyroid nodules show a larger total shift from 681.7 nm to 715 nm over the same thickness range, reflecting stronger optical path modulation due to higher refractive index. Sensitivity increases from 161 nm RIU^−1^ at 220 nm to a peak of 175 nm RIU^−1^ at 240–245 nm, then plateaus, while *Q*-factor peaks at 115.75 (230 nm) before dropping to 88.56 (240 nm) due to FWHM broadening to 8 nm. FOM follows suit, reaching 28.67 (230 nm) but declining at thicker layers. The optimal thickness window of 240–245 nm thus maximizes sensitivity.

**Table 3:** The performance parameters of our design with different defect thicknesses dd = 220 nm, 230 nm, 240 nm, 245 nm, at the crystalline phase of GST in the first stage

Material	Thickness (nm)	Peak wavelength (nm)	Refractive index (RIU)	Sensitivity (nm RIU^−1^)	*λ*_FWHM_ (nm)	*Q*	FOM (1/RIU)
Healthy at the crystallin phase of GST	220	654.1	2.020006	—	4.9	—	—
	230	665	2.020007	—	5.5	—	—
	240	678.5	2.020010	—	5.5	—	—
	245	685	2.020011	—	5.5	—	—
Thyroid nodules at crystallin phase of GST	220	681.7	2.191308	161	6	113.616	—
	230	694.5	2.191313	172	6	115.750	28.666
	240	708.5	2.191320	175	8	88.562	21.875
	245	715	2.191323	175	7.5	95.333	23.333

Table 4 presents the performance metrics of our design in the first stage, in the amorphous states of GST, across different defect layer thicknesses (220, 230, 240, and 245 nm). In the amorphous GST phase, the photonic crystal sensor displays a thickness-dependent red-shift of the defect mode, enabling fine-tuned spectral separation between healthy thyroid tissue and nodules. For healthy tissue (*n* ≈ 2.020), increasing defect-layer thickness from 220 nm to 245 nm shifts the resonance from 650 nm to 684 nm (Δ*λ* = 34 nm), with FWHM progressively widening from 4.3 nm to 5.5 nm and *Q*-factor declining from 151.16 to 124.36, reflecting reduced mode confinement at thicker layers. Thyroid nodules (*n* ≈ 2.191) exhibit a slightly larger total shift from 679 nm to 714 nm (Δ*λ* = 35 nm), maintaining a consistent 29–30 nm spectral gap from healthy tissue across all thicknesses. Sensitivity rises from 169 nm RIU^−1^ at 220 nm to a maximum of 175 nm RIU^−1^ at 240–245 nm, while FOM peaks at 29.96 (220 nm) and drops to 21.88 (240 nm) due to FWHM broadening up to 8 nm. The 240–245 nm thickness range emerges as optimal for nodule detection, thereby maximizing spectral resolution and diagnostic contrast in the amorphous phase—ideal for high-precision, label-free thyroid pathology screening *via* wavelength-based discrimination.

**Table 4:** The performance parameters of our design with different defect thicknesses dd = 220 nm, 230 nm, 240 nm, 245 nm, at the amorphous phase of GST in the first stage

Material	Thickness (nm)	Peak wavelength (nm)	Refractive index (RIU)	Sensitivity (nm RIU^−1^)	*λ*_FWHM_ (nm)	*Q*	FOM (1/RIU)
Healthy at amorphous phase of GST	220	650	2.020005	—	4.3	151.162	—
	230	664	2.020007	—	5	132.8	—
	240	677.5	2.020009	—	5.5	123.181	—
	245	684	2.020010	—	5.5	124.363	—
Thyroid nodules at amorphous phase of GST	220	679	2.191306	169	5.64	120.390	29.964
	230	693.5	2.191313	172	6.5	106.692	26.461
	240	707.5	2.191319	175	8	88.437	21.875
	245	714	2.191322	175	7.5	95.200	23.333

### 3.6 Effect of the incident angle on the transmission spectra of the “first stage” under amorphous and crystalline phases of GST material

In this section, the effect of a change in angle of incidence from 10° to 30° in steps of 10° corresponding to a TE polarized electromagnetic wave on the performance of the proposed biosensor has been studied. For this purpose, we have fixed the defect layer thickness to 240 nm.

**Fig. 8:**
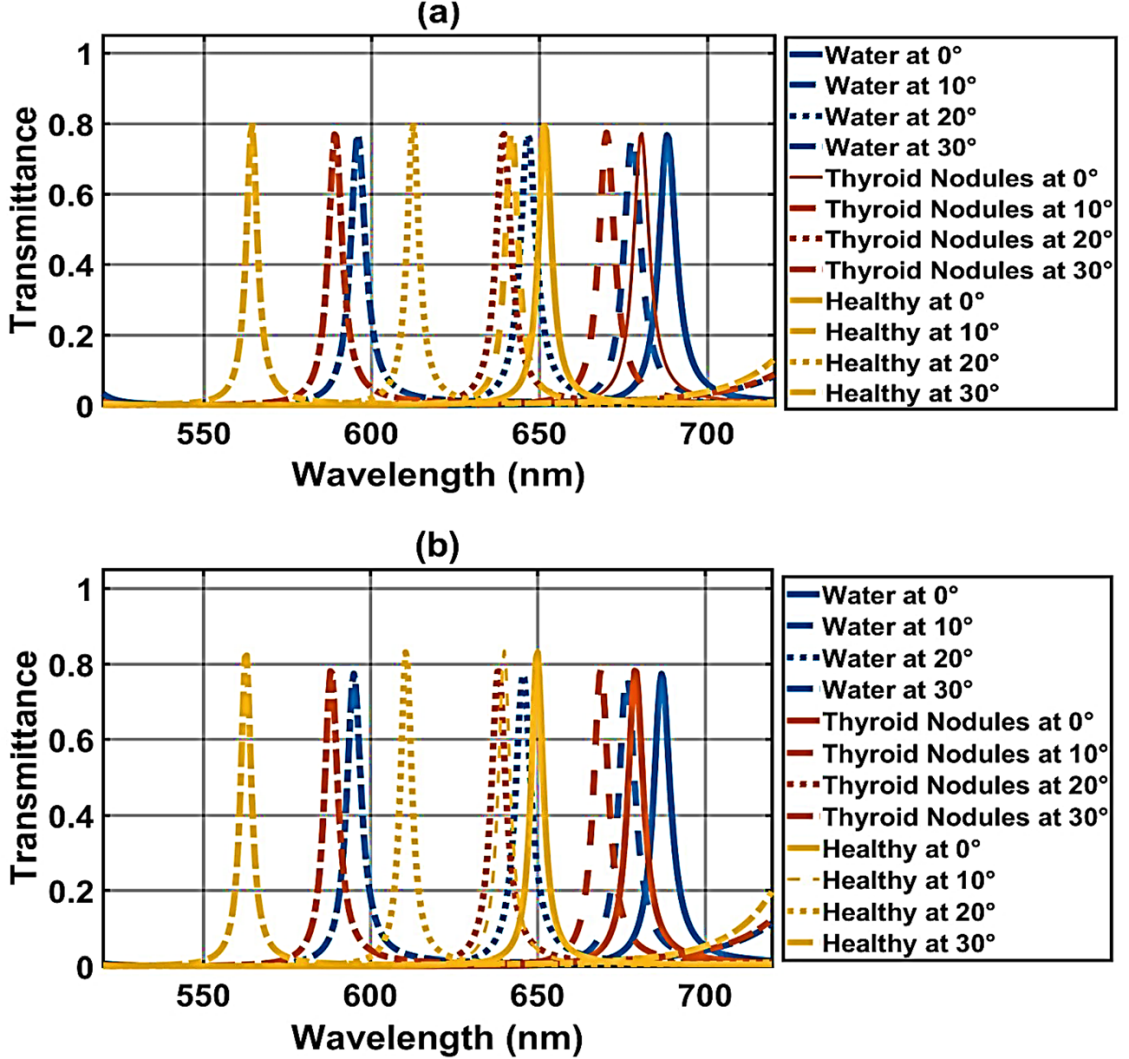
Transmission spectra of defect modes of the biosensor [Diamond/Silica)^3^ GDG (Diamond/Silica)^3^] loaded with healthy thyroid and thyroid nodules at dd = 240 nm and different angles *θ* = 0°,10°,20°,30° (a) under crystalline phase of GST an and (b) under amorphous phase of GST.

Fig. 8a and b shows the transmission spectra of the proposed structure under crystalline and amorphous phases of GST material at different incident angles, 10°, 20°and 30°. Under the crystalline phase of GST material, Fig. 8a shows that the increase in angle of incidence from 10° to 30° results in the blue shifting of defect modes. For example, the defect modes of thyroid nodules at an incident angle of 10° appear at 670 nm, but at a 30° incident angle, they appear at 589 nm.

Next, we switch the Ge_2_Sb_2_Te_5_ material from the CGST phase to the AGST phase, keeping all other structural parameters constant. Fig. 8b shows the transmittance spectra of the proposed structure under the amorphous phase of the GST material. Thus, the variation in angle of incidence can also be used to improve performance as well as confirmation of investigations carried out by the proposed design under CGST and AGST phases of Ge_2_Sb_2_Te_5_ material, which induces a reconfigurable property into our proposed photonic bio-sensing design.

Table 5 presents the performance of our design in the first stage, in the crystalline states of the phase change material for Healthy tissue and Thyroid Nodules across incident angles of 0°, 10°, 20°, and 30°. At a fixed 240 nm defect-layer thickness in the crystalline GST phase, the photonic crystal sensor exhibits a strong angle-dependent blue-shift of the defect mode with increasing incidence angle (0° → 30°), enabling angularly tunable spectral discrimination between healthy thyroid tissue and nodules. For healthy tissue (*n* ≈ 2.020), the resonance shifts from 678.5 nm at normal incidence (0°) to 587.5 nm at incidence angle 30°, with FWHM fluctuating between 4.5–6.0 nm narrowest at 30° (4.5 nm). Thyroid nodules (*n* ≈ 2.191) follow a parallel trend, blue-shifting from 708.5 nm at normal incidence (0°) to 613.5 nm at incidence angle 30°, preserving a consistent 29–30 nm spectral gap across all angles. Sensitivity decreases monotonically from 175 nm RIU^−1^ at 0° to 151 nm RIU^−1^ at 30°, while *Q*-factor remains stable (∼101–107). This angle-induced tunability with maintained pathological contrast and improved resonance sharpness at higher angles positions the sensor for multiplexed, angle-resolved biosensing, where 0° incidence angle offers maximum sensitivity for thyroid nodule detection.

**Table 5:** The performance of the proposed design (Diamond Silica)^3^ GDG (Diamond Silica)^3^ loaded with healthy and thyroid nodules at dd = 240 nm and different angles *θ* = 0°,10°,20°,30° under crystalline phases of GST

Material	Incident angles (θ°)	Peak wavelength (nm)	Refractive index (RIU)	Sensitivity (nm RIU^−1^)	*λ*_FWHM_ (nm)	*Q*	FOM (1/RIU)
Healthy at crystalline phase of GST	0	678.5	2.020010	—	5.5		—
	10	668.5	2.020008	—	6		—
	20	637.5	2.020003	—	5.5		—
	30	587.5	2.019995	—	4.5		—
Thyroid nodules at crystalline phase of GST	0	708.5	2.191320	175	7	101.214	25
	10	697.5	2.191315	169	6.5	107.307	26
	20	665.5	2.191300	169	6.5	102.384	26
	30	613.5	2.191277	151	6	102.25	25.166

The Table 6 outlines the performance of our design in the first stage, in the amorphous phase of GST for Healthy tissue and Thyroid Nodules across incident angles of 0°, 10°, 20°, and 30°. In the amorphous GST phase with thickness of defect layer 240 nm, the photonic crystal sensor demonstrates angle-dependent blue-shifting of the defect mode as the incidence angle increases from 0° to 30°, enabling dynamic spectral tuning while preserving clear pathological discrimination. For healthy thyroid tissue (*n* ≈ 2.020), the resonance shifts from 677.5 nm at normal incidence to 586.5 nm at 30°, with FWHM remaining narrow (4.5–5.5 nm) and peaking in sharpness at 30° (4.5 nm). Thyroid nodules (*n* ≈ 2.191) follow a nearly identical trend, blue-shifting from 707.5 nm at 0° to 612.5 nm at 30°, consistently maintaining a 29–30 nm spectral separation from healthy tissue across all angles. Sensitivity decreases steadily from 175 nm RIU^−1^ at 0° to 151 nm RIU^−1^ at 30°, reflecting reduced effective optical path modulation at oblique incidence. *Q*-factor fluctuates between 92.9 and 102.2, while FOM peaks at 25.1 at 20° before settling at 23.2 at 30°. This angle-tunable platform with stable diagnostic contrast, enhanced resonance sharpness at high angles, and minimal performance degradation offers a versatile, compact biosensing for real-time, label-free thyroid nodule detection through controlled angular interrogation.

**Table 6:** The performance of the proposed design (Diamond Silica)^3^ GDG (Diamond Silica)^3^ loaded with healthy and thyroid nodules at dd = 240 nm and different angles *θ* = 0°,10°,20°,30° under amorphous phases of GST

Material	Incident angles (*θ*°)	Peak wavelength (nm)	Refractive index (RIU)	Sensitivity (nm RIU^−1^)	*λ*_FWHM_ (nm)	*Q*	FOM (1/RIU)
Healthy at amorphous phase of GST	0	677.5	2.020009	—	5		
	10	667	2.020008	—	5.5		
	20	636.5	2.020003	—	5.5		
	30	586.5	2.019995	—	4.5		
Thyroid nodules at amorphous phase of GST	0	707.5	2.191319	175	7	101.071	25
	10	696.5	2.191314	172	7.5	92.866	22.933
	20	664.5	2.191300	163	6.5	102.230	25.076
	30	612.5	2.191276	151	6.5	94.230	23.230

Future improvements to the sensor performance could include optimizing the defect layer thickness and GST buffer configuration for higher field confinement, incorporating low-loss materials or anti-reflection coatings to reduce losses, or hybridizing with graphene for enhanced surface sensitivity. Additionally, increasing the number of periodic layers or employing asymmetric designs could further elevate the quality factor and sensitivity beyond the current values of 267 nm RIU^−1^ and 87.875, respectively.

### 3.7 Comparison with the previous research

Finally, Table 7 compares the performance of the proposed GST-based reconfigurable biosensor with recent photonic crystal and related sensing platforms [including new ref. 1–8]. While prior works achieve reasonable sensitivity, they generally lack active tunability. The key advance of this design is the incorporation of GST phase-change material as buffer layers, enabling reversible (amorphous-to-crystalline) tuning of the defect-mode resonance without structural changes or phase-matching requirements (unlike SPR-based devices). These results in superior sensitivity (up to 267 nm RIU^−1^ in crystalline phase) and dynamic adaptability, significantly enhancing discrimination capability for thyroid nodule detection compared to fixed-structure sensors.

**Table 7:** Comparison of the performance parameters of the proposed design with previous research work

Year	Sensitivity (nm RIU^−1^)	*Q*-factor	FOM (1/RIU)	Tunability	References
2019	25.75–51.49	Not mentioned	Not mentioned	No	29
2020	10	3 × 10^2^	15.1	No	30
2021	71–75	Not mentioned	Not mentioned	No	15
2024	224	Not mentioned	Not mentioned	No	52
2024	180–250	Moderate	∼100–200	Limited/No	69
2025	150–220	High	Moderate	Passive	70
2024	200–230	Not mentioned	Not mentioned	No	71
2024	210–260	Moderate	∼150	No	72
This work	175–267 (amorphous to crystalline)	(0.130–0.136) × 10^3^	(0.130–0.137) × 10^3^	Yes (reversible GST phase-change)	This work

## 4 Conclusion

In conclusion, this study presents a reconfigurable 1D defective photonic crystal biosensor incorporating Ge_2_Sb_2_Te_5_ (GST) phase-change material as buffer layers around the cavity defect in the structure (Diamond/Silica)^3^ GDG (Diamond/Silica)^3^. The reversible phase transition of GST between amorphous and crystalline states enables active tuning of the defect mode resonance in the visible range.

Key findings include a high sensitivity of 267 nm RIU^−1^ and quality factor of 87.875 in the crystalline GST phase at normal incidence and 220 nm defect thickness, outperforming previous photonic crystal biosensors in tunability and spectral selectivity. These results demonstrate the potential of GST-based designs for label-free, accurate detection of malignant thyroid nodules, paving the way for compact and practical photonic biosensing platforms in clinical diagnostics. Most importantly, the results confirm that lyophilized plasma provides significantly better discrimination between malignant and benign thyroid nodules than liquid plasma, due to the removal of dominant water absorption and exposure of subtle biomolecular dielectric differences.

Future scope includes integrating the proposed GST-based photonic biosensor with automated THz platforms or microfluidic systems for rapid, point-of-care analysis of plasma samples. Such developments could enable real-time, high-throughput screening and further bridge the gap between theoretical simulations and routine clinical diagnostics for thyroid malignancies.

## Ethical statement

We should disclose here that we don't use any human samples—blood chemicals or otherwise—because we work in the simulation and computational sectors. Only publicly available tables and data are used.

## Author contributions

W. N and A. H. A. conceived the main idea and the designed structure. W. N. F. S., S. E., S. S and A. H. A designed and conducted the analyses and software. Also, W. N. F. S., S. E., S. S and A. H. A analyzed the results. All authors have reviewed the manuscript.

## Conflicts of interest

The authors declare no competing interests.

## Data Availability

The datasets used and analyzed in this study are available upon reasonable request from the corresponding author.
